# Giant Cell Tumor of the Proximal Fibula With Common Peroneal Nerve Neuropraxia

**DOI:** 10.7759/cureus.32984

**Published:** 2022-12-27

**Authors:** Rohan Chandanwale, Aditya Pundkar, Ajay Chandanwale, Kashyap Kanani, Rameez Bukhari, Ankit Mittal

**Affiliations:** 1 Department of Orthopaedics, Jawaharlal Nehru Medical College, Datta Meghe Institute of Medical Sciences, Wardha, IND; 2 Department of Orthopaedics, Jawaharlal Nehru Medical College, Datta Meghe Institute Of Medical Sciences, Wardha, IND; 3 Department of Orthopaedics, B. J. Government Medical College, Pune, IND

**Keywords:** galvanic stimulation, wide excision, common peroneal nerve, proximal fibula, giant cell tumour

## Abstract

Giant cell tumor (GCT) is among the commonest benign tumors and represents 5% of bone neoplasms. It is more common in young adults aged between 20 and 40 years. The distal femur is one of the most common sites, with the proximal tibia and distal radius the next frequently involved site, respectively. Previous research indicates that the tumor is an uncommon occurrence at this given age and location. Surgical management is the primary treatment for GCT universally. Extended curettage with the use of an argon beam cauterizer, a power burr, bone cement, hydrogen peroxide, phenol, liquid nitrogen, and zinc chloride are some of the treatment modalities for GCT. Opting for appropriate surgical treatments plays a crucial role to reduce the rate of recurrence and improve functional and oncological outcomes. In this case study, a 55-year-old male was diagnosed with GCT of the head of the right fibula with foot drop. The patient was managed with wide excision of the tumor and anchoring of lateral collateral ligament and biceps femoris to medial tibia condyle followed by postoperative galvanic stimulation for common peroneal nerve neuropraxia and guarded weight-bearing mobilization with bracing for knee joint. After 12 months of follow-up, there is no evidence of recurrence with a stable knee joint and dorsiflexion of the right ankle up to the neutral position.

## Introduction

Giant cell tumor (GCT) is among the commonest benign tumors and represents 5% of bone neoplasms. It is more common in young adults aged between 20 and 40 years [1]. The distal femur is one of the most common sites, with the proximal tibia and distal radius the next frequently involved sites, respectively [2]. GCT is aggressive locally with a high recurrence rate and is classified under osteoclastic giant cell-rich tumors. Its local aggressiveness can range in intensity from isolated symptoms brought on by cortical damage to metastasis and growth in nearby soft tissue [3]. GCT that develops within the axial skeleton is typically unresectable and can result in local complications. GCTs are often solitary lesions, although 1%-2% of them may be multicentric, which might indicate the presence of several main lesions or only bone metastases from a single primary lesion [4]. Histologically, there are three different types of cells: GCT stromal cells of osteoblastic origin, mononuclear histiocytic cells, and multinucleated giant cells of an osteoclast-monocyte lineage [5]. The giant cells are mainly responsible for the tumor's resorption of bone. Monocytes are attracted by the spindle-shaped stromal cells, which encourage the cells to coalesce into massive cells. Additionally, the stromal cells augment the giant cells' capacity for resorption [6]. Swelling and pain around the involved joints are the main signs of this condition, followed by a restricted motion of the involved joint and occasionally pathological fractures. Histological and radiographic results serve as the foundation for the final diagnosis. Surgical management is the primary treatment for GCT universally. Extended curettage with the use of an argon beam cauterizer, a power burr, bone cement, hydrogen peroxide, phenol, liquid nitrogen, and zinc chloride are some of the treatment modalities for GCT. Opting for appropriate surgical treatments plays a crucial role to reduce the rate of recurrence and improving functional and oncological outcomes [7].

## Case presentation

**Figure 1:**
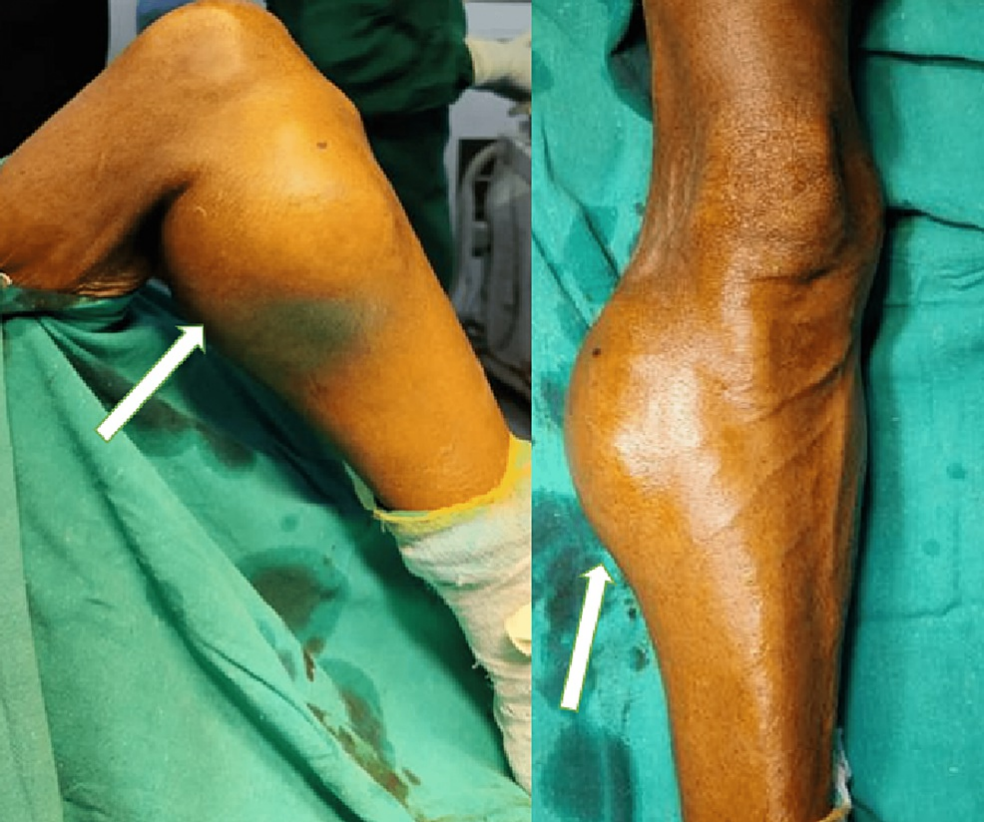
Preoperative clinical site of the tumor (anteroposterior and lateral view of the knee).

A 55-year-old male came to the Orthopaedic Outpatient Department with complaints of pain and swelling over the right proximal leg for six months and weakness in the right foot for two months. The pain was localized, gradual in onset, dull aching in character, and aggravated on knee movements. Four months after the onset of symptoms, the patient gradually developed weakness in the right foot, which progressed as the swelling increased in size. Examination showed diffuse swelling over the lateral aspect of the right proximal leg with the presence of dilated veins over the overlying tense and shiny skin (Figure 1). The distal pulsations of the dorsalis pedis and posterior tibial artery were palpable in the affected limb.

**Figure 2:**
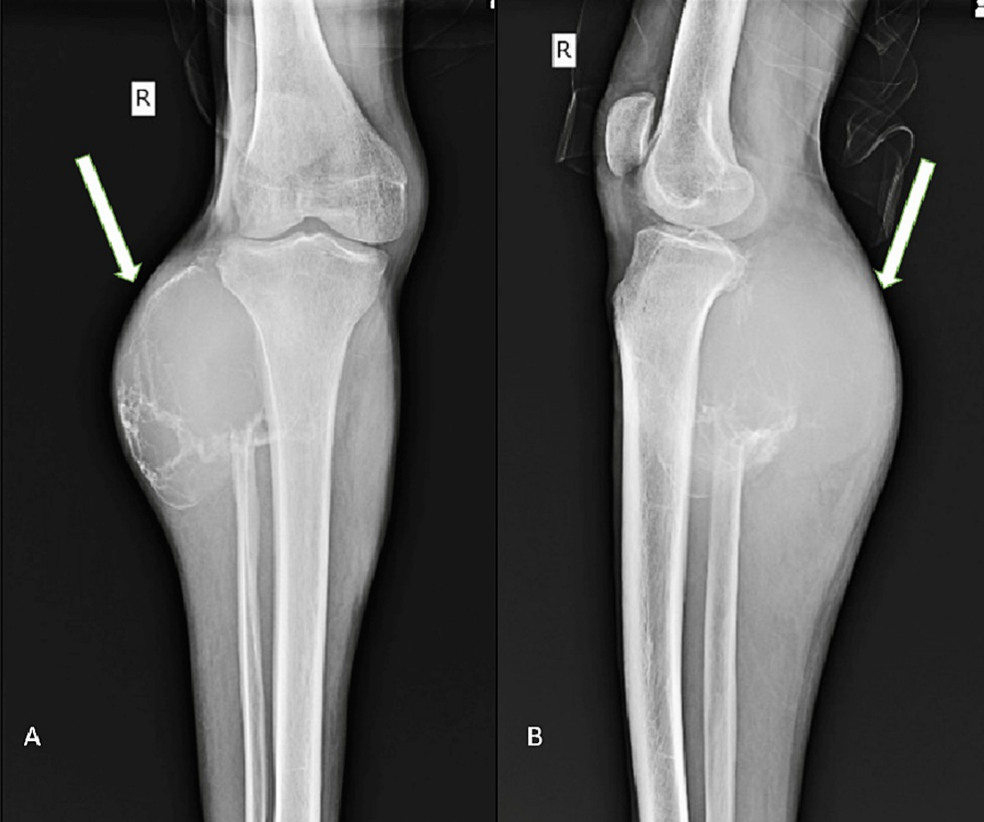
Preoperative X-ray of the tumor. (A) Anteroposterior view of the knee. (B) Lateral view of the right knee.

There was a weakness of the dorsiflexors of the right foot with grade 1 power preoperatively. There was hypoesthesia over the anterolateral aspect of right lower leg and dorsum of the foot (L4, L5, and S1 dermatome) with normal deep tendon and superficial reflexes. X-ray showed a large, poorly defined lytic lesion with cortical thinning of the proximal metaphyseal region of fibula (Figure 2).

**Figure 3:**
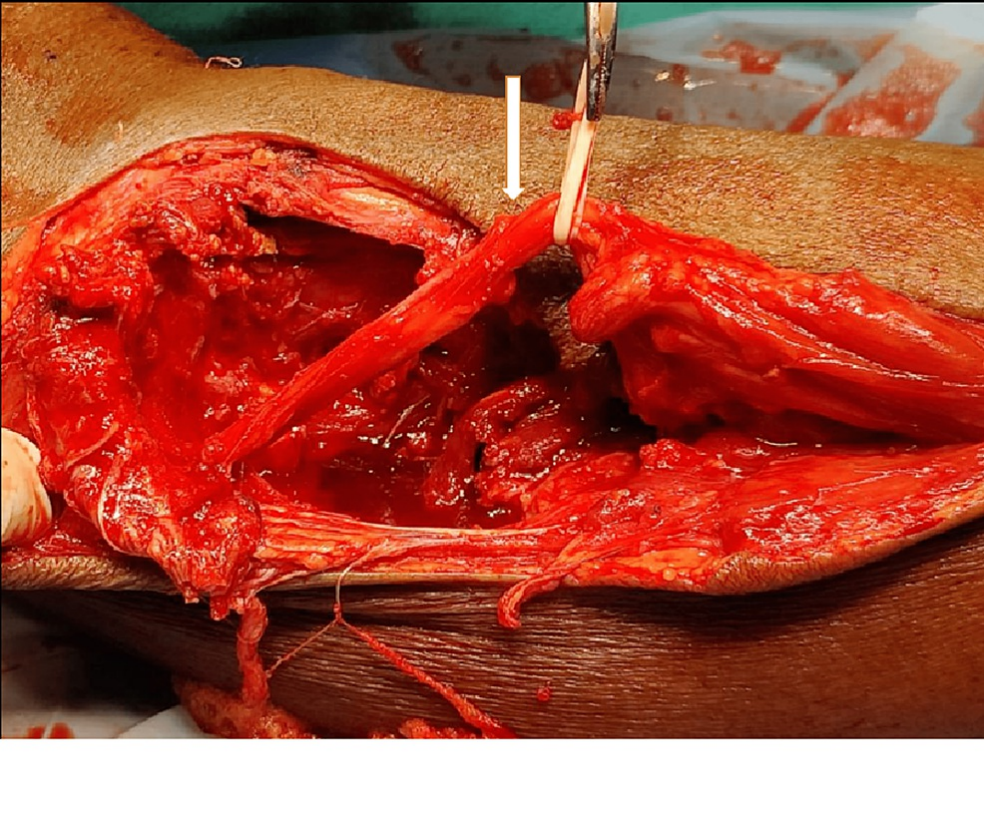
Intra operative picture of intact common peroneal nerve

**Figure 4:**
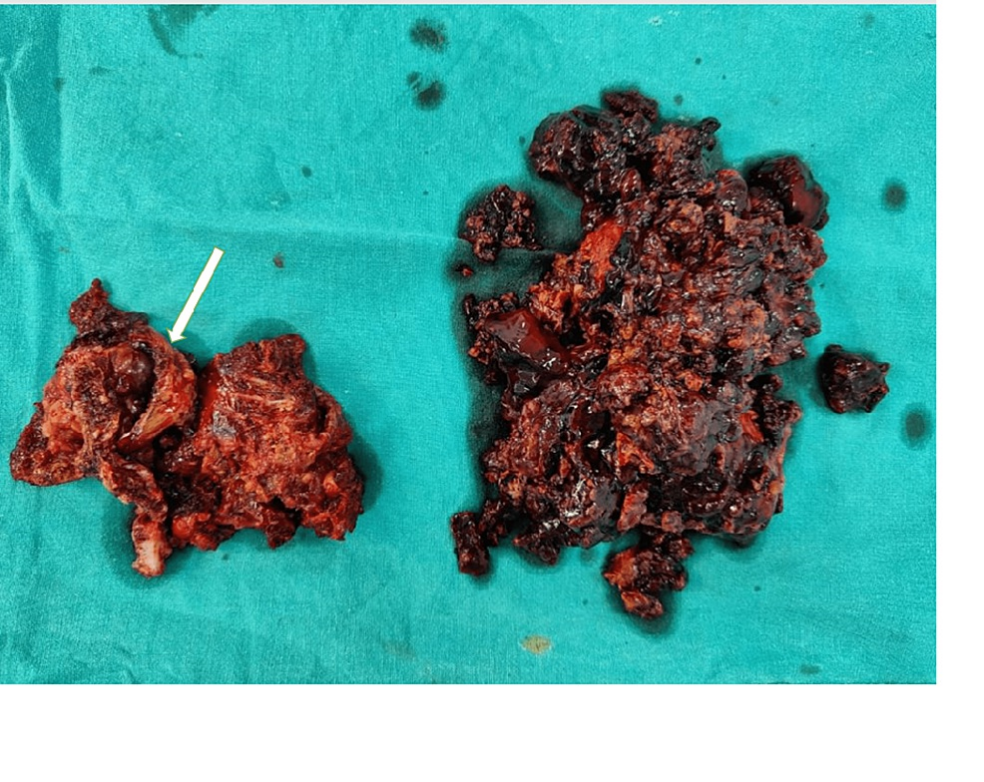
Clinical picture of the excised tumor with a thin cortex.

Blood parameters were found to be normal. A core needle biopsy was obtained and was suggestive of a giant cell tumor. The patient was explained about the treatment for complete excision of the tumor along with excision of the fibular head. The patient was managed with wide excision of the tumor. Intraoperatively, the common peroneal nerve was found to be intact. The lateral collateral ligaments and biceps femoris were anchored at the proximal tibia with nonabsorbable sutures (Figures 3-4).

**Figure 5:**
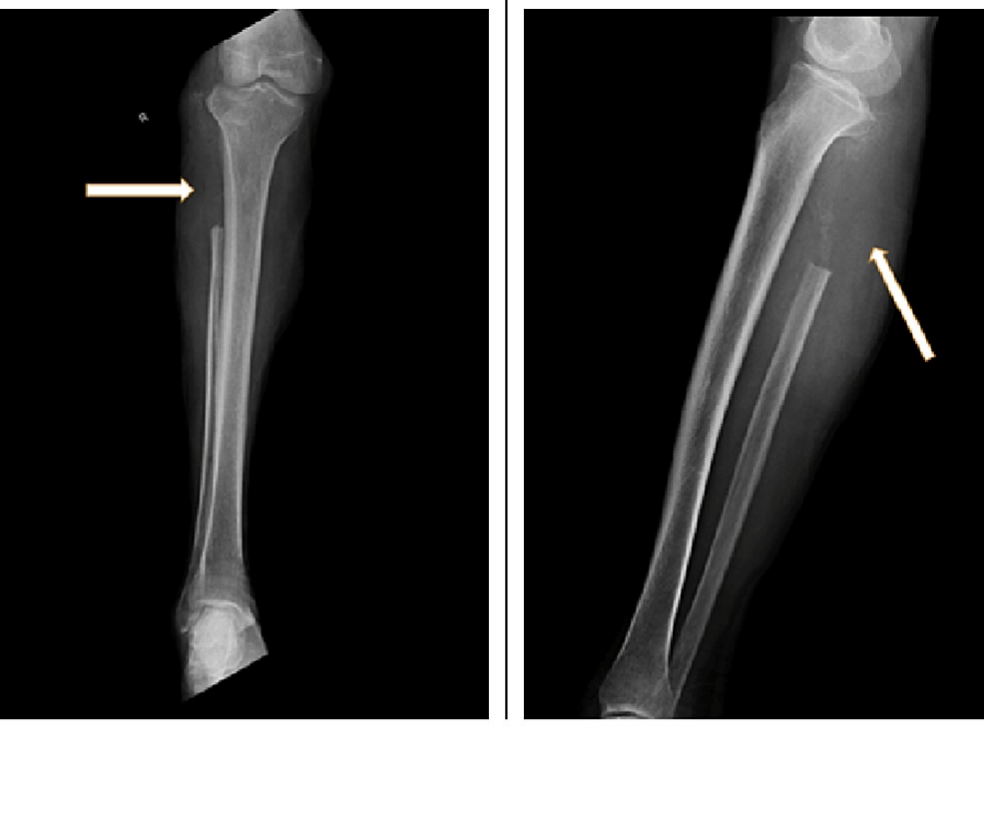
Twelve months follow-up X-ray showing no evidence of local recurrence.

The histopathological image was suggestive of many giant cells, with multiple nuclei dispersed among oval to spindle cells without atypia or mitotic features, thus confirming the diagnosis of a GCT. Postoperatively patient was kept on supportive bracing and gradual weight bearing as tolerated by the patient. Adjuvants such as denosumab, bisphosphonates, and interferon alfa (IFNa) were not used. The patient underwent a series of galvanic stimulation for common peroneal nerve neuropraxia, and the power of dorsiflexors of the right foot improved from grade 1 to grade 3, with dorsiflexion of the right foot up to the neutral position and improvement in hypoesthesia over anterolateral aspect of lower leg and dorsum of foot over three months. After 12 months of follow-up, there is no evidence of recurrence at the local site (Figure 5).

## Discussion

GCT should be well treated as it is a benign tumor with localized aggressiveness. Population aged between 20 and 40 years have a higher incidence, with the distal femur and proximal tibia being the common sites [8-10]. In our case, a 55-year-old male was detected with a GCT of the head of the right fibula. Previous research indicates that the tumor is an uncommon occurrence at this given age or location. Pujani et al. described a case of GCT around the knee joint in a 55-year-old misdiagnosed as a malignant sarcoma [11]. GCT has a high tendency to recur after surgical removal as it is locally aggressive [7,12]. A retrospective study done in China performed in multiple centers concluded that there was a 23.4% recurrence rate of the GCT in the 29- to 30-year-old population, with a higher recurrence rate in the proximal fibula compared to the distal femur in terms of its location [13]. The selection of appropriate treatment is very important to reduce the recurrence rate. Complete removal of the tumor and preserving the surrounding soft tissue structures to maintain the functional outcome is the aim of the treatment [14,15]. Many studies suggest that treating the GCT with wide local excision has a low recurrence rate. Klenke et al. found a decreased recurrence rate of 5% and 25% of patients managed with wide local excision and intralesional surgery, respectively [16]. A study by Willand et al. concluded with a discussion of recent developments in the field of *Electrical Stimulation to Promote Peripheral Nerve Regeneration*. The current literature suggests that low-frequency electrical stimulation will soon be used therapeutically to effectively encourage the regeneration of axons after surgical repair, as the stimulation has given promising results to maximize recovery in various peripheral nerve injuries [17].

## Conclusions

A GCT has a high postoperative recurrence rate as it is locally aggressive. An excellent functional and oncological prognosis is achieved with wide local excision of the tumor along with preservation of the common peroneal nerve and anchoring of the biceps femoris and lateral collateral ligament to the lateral tibial condyle. Postoperative galvanic stimulation for common peroneal nerve neuropraxia added to the improvement of functional outcomes. 
